# Effects of the timing of acute mulberry leaf extract intake on postprandial glucose metabolism in healthy adults: a randomised, placebo-controlled, double-blind study

**DOI:** 10.1038/s41430-023-01259-x

**Published:** 2023-01-17

**Authors:** Masaki Takahashi, Yui Mineshita, Jumpei Yamagami, Chunyi Wang, Kyoko Fujihira, Yu Tahara, Hyeon-Ki Kim, Takashi Nakaoka, Shigenobu Shibata

**Affiliations:** 1 Institute for Liberal Arts, Tokyo Institute of Technology, Meguro, Tokyo 152-8550 Japan; 2 School of Environment and Society, Tokyo Institute of Technology, Meguro, Tokyo 152-8550 Japan; 3 School of Advanced Science and Engineering, Waseda University, Shinjuku, Tokyo 162-8480 Japan; 4 Functional Food Research Institute, FANCL Research Institute, Totsuka, Kanagawa 244-0806 Japan; 5 Japan Society for the Promotion of Science, Chiyoda, Tokyo 102-0083 Japan; 6 Graduate School of Biomedical and Health Sciences, Hiroshima University, Hiroshima, 734-0037 Japan; 7 Japan Organization of Occupational Health and Safety, Kawasaki, Kanagawa 211-0021 Japan

**Keywords:** Randomized controlled trials, Nutrition

## Abstract

**Background/Objectives:**

Glucose tolerance is controlled by the internal clock and is worse in the evening. From a chrononutrition perspective, diabetes prevention requires evaluating the antidiabetic effects of the timing of functional ingredients and nutrient intake. The purpose of this study was to investigate the timing effects of acute mulberry leaf extract (MLE) intake on postprandial glucose levels in young adults.

**Subjects/Methods:**

Twelve young adults underwent four trials. Blood samples were collected in a fasting state and at 30, 60, 120, and 180 min after eating a mixed meal. The study had a randomised, placebo-controlled, double-blind trial design involving: (1) morning placebo trial (08:00 h; MP trial), (2) evening placebo trial (18:00 h; EP trial), (3) morning MLE trial (08:00 h; MM trial), and (4) evening MLE trial (18:00 h; EM trial).

**Results:**

The incremental area under the blood glucose curve (iAUC) in the EM trials was significantly lower than that in the EP trials (*P* = 0.010). The postprandial glucose concentrations 120 min after the meal were significantly lower in the EM trials than those in the EP trials (*P* = 0.006). The postprandial insulin concentrations at 120 min were significantly lower in the MM trials than those in the MP trials (*P* = 0.034). Moreover, the postprandial insulin concentrations 180 min after the meal were significantly lower in the EM trials than those in the EP trials (*P* = 0.034).

**Conclusions:**

MLE intake in the evening, but not in the morning, was effective in improving glucose tolerance.

**Trial registration:**

Clinical trial reference: UMIN 000045301; website of trial registry: https://center6.umin.ac.jp/cgi-open-bin/ctr/ctr_view.cgi?recptno=R000051340.

## Introduction

Postprandial glucose elevation and prolonged postprandial hyperglycaemia are risk factors for diabetes [1–3]. It has been reported that glucose levels differ according to various dietary and physiological factors such as volume and content of the meal [4, 5]. Recently, chrononutrition, which considers the timing of food intake in the day, has been gaining attention in nutritional research as translational research of chronobiology [6, 7]. In this research field, many studies have reported that glucose tolerance is worse during the latter part of the day [8–12]. These differences are caused by the circadian clock-regulated physiological function system, including digestion, absorption, metabolism, and endocrine function in some tissues, such as the stomach, intestines, and liver.

Mulberry leaf extract (MLE), which contains 1-deoxynojirymycin (DNJ), has blood glucose-lowering and antidiabetic properties [13]. Several animal and human studies have shown that the intake of MLE containing DNJ suppresses postprandial glucose elevation [14–16]. Regarding the glucose-lowering effects of MLE in humans, Asai et al. [17] reported that ingestion of MLE with 3, 6, or 9 mg of DNJ, attenuated postprandial glucose elevation, depending on the dose. Nakagawa et al. [18] found that oral MLE intake (6.3 mg of DNJ) was absorbed into the blood with peak levels after 1.5 h. Considering the absorption and bioactivity of DNJ, acute MLE intake containing more than 6.0 mg of DNJ with a test meal has antidiabetic effects, such as glucose-lowering and regulating insulin secretion.

A possible mechanism to explain the glucose-lowering effects of DNJ intake has been suggested; DNJ acts as an inhibitor of intestinal α-glucosidase by inhibiting the binding of substrates to the enzyme [19], which inhibits glucose absorption in the small intestine and increases glucose metabolism in the liver [14]. Another factor contributing to the antidiabetic effects of DNJ intake is the improvement in insulin function and sensitivity. Previous research has shown that MLE, including DNJ, reduces insulin concentrations in healthy and hyperglycaemic subjects [15, 20]. However, no information is available regarding the effects of timing MLE intake on postprandial glucose levels. To prevent diabetes and considering the concept of chrononutrition, it is important to consider the antidiabetic effects of timing of ingredient and nutrient intake. This could provide new evidence regarding the timing of antidiabetic food intake, especially MLE, from a chrononutrition perspective.

In this study, we examined the timing effects of acute MLE intake on postprandial glucose levels in healthy adults. We hypothesised that the glucose-lowering effects of MLE may be greater in the evening because glucose tolerance is worse in the evening.

## Subjects and methods

### Subjects

Participants were recruited from Tokyo Institute of Technology and Waseda University via word of mouth and study posters on display on the campus. Twelve (male; *n* = 8, female; *n* = 4) healthy young adults (aged 21–39 years) participated in this study. The exclusion criteria were as follows: (1) diagnosed with diabetes or dyslipidaemia; (2) taking any antioxidant, anti-obesity, or antidiabetic medication or supplements; (3) taking depression, sleeping, or steroid medications; (4) obese (body mass index [BMI] > 35 kg/m^2^) or suffering from sleep apnoea; (5) smoker; (6) set pacemaker or metal in the body (excluding dental fillings); and (7) engaged in shift-work or travel with jetlag within the previous 2 weeks. To rule out the presence of any of these exclusion criteria, all participants completed a health-related questionnaire about dietary intake and physical activity, including exercise, prior to the study (Fig. S1). This study was conducted in accordance with the guidelines of the Declaration of Helsinki and was approved by the ethics committee of the Tokyo Institute of Technology (Approval No. 2021080). All participants provided informed consent prior to enroling in the study. This clinical trial was registered under the local institutional board of the University Hospital Medical Information Network, Japan (Clinical Trial reference: UMIN 000045301).

### Study design and procedure

**Fig. 1:**
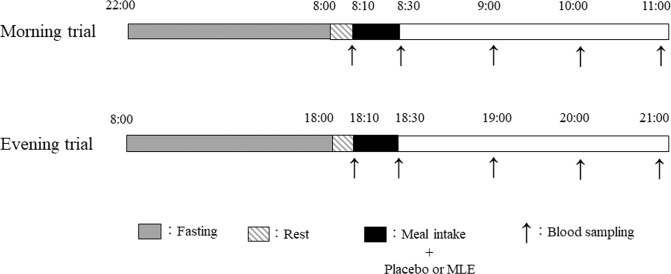
Protocols for the morning and evening trials. Gray box: fasting duration, Diagonal box: resting duration, Black box: meal and placebo or MLE intake, Up arrow: blood sampling.

The trials were performed at the Tokyo Institute of Technology between August and November 2021. A randomised, placebo-controlled, double-blind, counterbalanced crossover design was used. Each participant participated in four trials in a randomised order: (1) morning placebo trial (08:00; MP trial), (2) evening placebo trial (18:00; EP trial), (3) morning MLE trial (08:00; MM trial), and (4) evening MLE trial (18:00; EM trial; Fig. 1). Randomisation was achieved using computer-generated random numbers using Excel software. An independent researcher who was not involved in this study generated the sequence. The interval between trials was at least 1 week. In all the trials, we asked the participants to maintain their usual daily sleep and wake cycle. For the MP and MM trials, all participants were required to visit the laboratory at 08:00 h after a minimum of 10-h overnight fast (no intake of any food or beverage, except water). The dinner on the previous day was provided in both morning trials. For the EP and EM trials, all participants were required to visit the laboratory at 18:00 after a minimum 10-h fast (no intake of any food or beverage, except water) maintaining a resting state. Breakfast on the day of the experiment was provided in both evening trials. In this study, fasting duration was almost the same as in previous studies, which have reported that fasting duration influences glucose tolerance and metabolism [8, 21]. After a 10 min rest, a fasting venous blood sample was collected by venipuncture while the participants were in a seated position. Venous blood samples were collected 30, 60, 120, and 180 min after the initiation of the test meal for each trial.

### Test meals

The test meals used in all the trials were purchased as a set meal. Although we did not evaluate the amount of ingredients in each meal, these meals were almost identical. In addition, the test meals were provided as mixed meals consisting of rice, tofu, onions, cheese, mushrooms, bacon, Chinese cabbage, pork ham, carrots, potatoes, tomatoes, macaroni, and celery. The mixed meal was prepared according to body mass (average: 60 kJ/kg body mass) by a registered dietitian. The energy content of the meal was 15% from fats, 70% from carbohydrates, and 15% from proteins. Previous studies have reported that this macronutrient composition increases postprandial glucose in healthy adults [22, 23]. All participants were asked to consume the test meal within 20 min. The time taken to consume the mean in the first trial was recorded and replicated in subsequent trials. The participants consumed either a placebo (cellulose, 6 tablets, 1 mg per tablet, total 6 mg, no DNJ) or MLE supplements (DNJ, 6 tablets, 1 mg per tablet, total 6 mg) with breakfast or dinner. It has been reported that this amount of DNJ decreases postprandial blood glucose levels [17]. During each trial, subjects sat in a chair (reading, writing, or using an electronic device such as PC or smart phone) except when eating the mixed test meal. In addition, they did not eat other food or drink except water after eating the mixed test meal.

### MLE and placebo content

The placebo (cellulose, 1 mg per tablet, no DNJ) and MLE (DNJ, 1 mg per tablet) tablets used in this study were provided by FANCL Corporation (Yokohama, Japan). Each tablet contained 1% calcium stearate to fill the tablets.

### Baseline measurements of body mass, body fat, and muscle mass

For all participants, body mass, body fat, fat-free mass, and muscle mass were measured to the nearest 0.1 kg using a digital scale (Inbody 230, Inbody Inc., Tokyo Japan) in a fasting state. We used the data taken at the first trial as the baseline measurement. BMI was calculated as weight in kilograms divided by the square of height in metres.

### Blood sampling and biochemical assays

To measure serum blood markers such as insulin, triacylglycerol (TAG), and non-esterified fatty acids (NEFAs), samples were allowed to clot for 30 min at room temperature and then centrifuged at 3000 rpm for 10 min. The serum sample was dispensed into plain microtubes and stored at −80 °C until the assay was performed. For plasma glucose measurements, venous blood samples were collected in tubes containing sodium fluoride-ethylenediaminetetraacetic acid. For the measurement of plasma gastric inhibitory polypeptide (GIP) and glucagon-like peptide 1 (GLP-1), venous blood samples were collected in tubes containing a DPP-4 inhibitor and protease inhibitor cocktail (BD, Tokyo, Japan). Thereafter, both samples were immediately centrifuged and stored at −80 °C until analysis. Enzymatic colorimetric assays were used to measure the plasma concentrations of glucose (GLU-HK (M); Shino-test Corporation, Kanagawa, Japan), serum TAG (Pure Auto S TAG-N; Sekisui Medical Company Limited, Tokyo, Japan), and serum NEFAs (Wako Pure Chemical Industries Limited). Enzyme-linked immunosorbent assays were used to measure plasma concentrations of insulin (Mercodia Insulin ELISA; Mercodia AB, Uppsala, Sweden), active GIP, and active GLP-1 (Yanaihara Institute, Inc. ELISA; Yanaihara Institute, Inc. Shizuoka, Japan).

### Statistical analysis

Data were analysed using Predictive Analytics Software, version 28.0 for Windows (SPSS, Inc., IBM, Tokyo, Japan). The primary outcome measure was the incremental area under the blood glucose curve (iAUC) of plasma glucose, and the secondary outcome measure was postprandial glucose concentrations. The sample size was estimated using G*Power 3.1 and data from previous studies on the effects of timing of meals on postprandial glucose [22, 23]. A sample size of 11 was determined to have approximately 80% power to detect changes in glucose-lowering effects at a significance level of 0.05. The Kolmogorov–Smirnov test was used to check for the normality of the distribution of all blood parameters. Paired *t*-tests were used to compare between the MP and EP trials on the iAUC of blood biomarkers. To compare the effects of the timing of diet (morning and evening) on postprandial blood parameters, a two-factor ANOVA with repeated measures was used to determine the effect of meal timing (morning or evening) and postprandial interval time (0–180 min) on blood markers. Moreover, a two-factor ANOVA with repeated measures was used to determine the effect of the trial (placebo or MLE) and intake timing (morning or evening) on the iAUC of blood markers, which was calculated using the trapezoidal rule. To evaluate the effects of timing of acute MLE intake on postprandial blood markers, a three-factor ANOVA with repeated measures was used to determine the effect of the trial (placebo or MLE), meal intake timing (morning or evening), and postprandial interval time (0–180 min) on the blood marker concentrations. When significant main or interaction effects were detected, the Bonferroni method was used for post-hoc comparisons. The *P*-values reported for the comparisons of postprandial blood parameters at each time are subsequent to the Bonferroni correction. Statistical significance was set at *P* < 0.05. Results were presented as means with standard errors.

## Results

### Baseline physical characteristics and fasting blood biomarker concentrations

The physical characteristics and baseline blood biomarker concentrations of the participants are summarised in Table 1.

**Table 1:** Physical characteristics and baseline fasting blood biomarker concentrations of the subjects.

Age (years)	29.8 ± 2.2
Height (cm)	166.3 ± 2.5
Body mass (kg)	59.9 ± 3.6
BMI (kg/m^2^)	21.5 ± 0.9
Body fat (%)	21.2 ± 2.4
Fat-free mass (kg)	46.9 ± 2.8
Muscle mass (kg)	26.0 ± 1.7
Glucose (mmol/l)	4.9 ± 0.1
Insulin (mu/l)	7.4 ± 0.8
GIP (pg/ml)	14.2 ± 2.8
GLP-1 (pg/ml)	7.9 ± 1.2
TAG (mmol/l)	0.9 ± 0.1
NEFA (mmol/l)	0.7 ± 0.1

All data are presented as mean ± SE.

### Effects of meal timing on postprandial blood biomarker concentrations

**Fig. 2:**
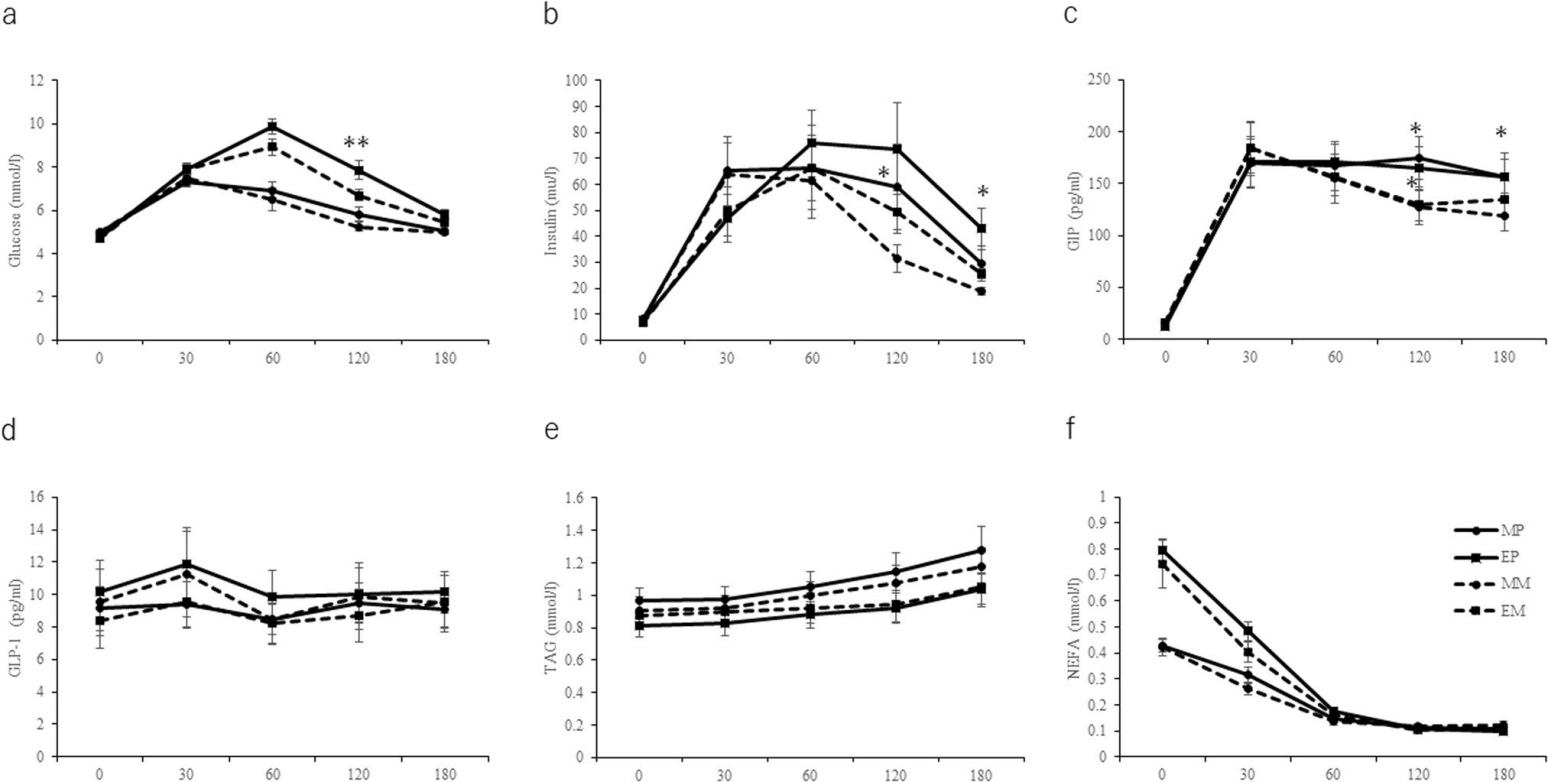
Fasting and postprandial blood biomarker concentrations. Fasting and postprandial plasma concentrations of glucose (**a**), insulin (**b**), GIP (**c**), GLP-1 (**d**), TAG (**e**), and NEFA (**f**) in the morning and evening trials. (Morning placebo (MP), evening placebo (EP), morning MLE (MM), and evening MLE (EM)). The values are presented as means while standard errors are represented by bidirectional bars. The mean values are significantly different from those of MLE trials at same mealtime: *P < 0.05, **P < 0.01 with Bonferroni correction. Abbreviations: MLE mulberry leaf extract, MM morning mulberry leaf extract, MP morning placebo, EM evening mulberry leaf extract, EP evening placebo, GIP gastric inhibitory polypeptide, GLP-1 glucagon-like peptide 1, NEFA non-esterified fatty acid, TAG triacylglycerol.

The iAUC of plasma glucose are shown in Table 2. The iAUC of plasma glucose in the evening trials was higher than that in the morning trials (*P* = 0.001). The plasma glucose concentrations are shown in Fig. 2a. A two-factor ANOVA revealed significant main effects (*P* = 0.001), time (*P* = 0.001), and timing × time interaction (P = 0.001) of the trial on plasma glucose concentrations. Post-hoc tests revealed that the glucose concentrations in the evening trials at 30 (*P* = 0.040), 60 (*P* = 0.001), 120 (P = 0.008), and 180 (*P* = 0.025) min after meal intake were significantly higher than those in the morning trials. Serum insulin concentrations are shown in Fig. 2b. A two-factor ANOVA revealed significant main effects of time (*P* = 0.001) and a timing × time interaction (*P* = 0.004) on serum insulin concentrations. Post-hoc tests revealed that the insulin concentrations in the morning trials were significantly higher than those in the evening trials at 30 min (*P* = 0.006) after meal intake. Plasma GIP concentrations are shown in Fig. 2c. A two-factor ANOVA revealed significant main effects of time (*P* = 0.001) on plasma GIP concentrations. In addition, for plasma GLP-1 concentrations, a two-factor ANOVA revealed no significant main effects or interactions (Fig. 2d). Serum TAG concentrations are shown in Fig. 2e. A two-factor ANOVA revealed significant main effects of time (*P* = 0.001) on serum TAG concentrations. The iAUC of serum NEFA concentrations in the evening trials was higher than that in the evening trials (*P* = 0.001, Table 2). Moreover, for serum NEFA concentrations, a two-factor ANOVA revealed significant main effects of timing (*P* = 0.001), time (*P* = 0.001), and a timing × time interaction (*P* = 0.001, Fig. 2(f)). Post-hoc tests revealed that the concentrations of NEFA at 30 min (*P* = 0.007) after meal intake were significantly higher in the evening trials than in the morning trials.

**Table 2:** The iAUC of blood biomarkers in each trial.

	MP (*n* = 12)	MM (*n* = 12)	EP (*n* = 12)	EM (*n* = 12)	*P* (trial)	*P* (time)	*P* (Interaction)
Glucose (mmol/3 h/l)	1107 ± 46^###^	1057 ± 42	1397 ± 47**	1275 ± 43	0.003	0.001	0.003
Insulin (mU/3 h/l)	9491 ± 1989	7239 ± 1266	10,655 ± 2054	8305 ± 1481	0.007	0.112	0.932
GIP (pg/3 h/ml)	28,019 ± 3526	23,983 ± 2950	27,550 ± 2864	24,663 ± 3037	0.002	0.829	0.599
GLP-1 (pg/3 h/ml)	1641 ± 202	1738 ± 305	1856 ± 296	1591 ± 265	0.333	0.694	0.083
TAG (mmol/3 h/l)	198 ± 19	186 ± 17	163 ± 15	170 ± 18	0.803	0.011	0.489
NEFA (mmol/3 h/l)	32 ± 2^###^	31 ± 3	44 ± 3	40 ± 4	0.266	0.002	0.493

All data are presented as means ± SEs.

The mean values are significantly different from those of evening trials, ^###^*P* < 0.001; The mean values are significantly different from that of MLE trials at same meal time, **P < 0.01 with Bonferoni correction.

*MP* morning placebo, *EP* evening placebo, *MM* morning MLE, *EM* evening MLE.

### Effects of MLE intake on postprandial blood biomarker concentrations

The iAUC in the EM trials was significantly lower than that in the EP trials (*P* = 0.010). A three-factor ANOVA revealed a significant timing × time interaction (*P* = 0.004) and trial × time interaction (*P* = 0.001). Post-hoc tests revealed that the glucose concentrations in the EM trials were significantly lower than those in the EP trials at 120 min (*P* = 0.006) after meal intake. Three-factor ANOVA analysis revealed a significant timing × time interaction (*P* = 0.004) and trial × time interaction (*P* = 0.001) in serum insulin concentrations. Post-hoc tests revealed that the insulin concentrations in the MM trials at 120 min (*P* = 0.034) were significantly lower than those in the MP trials after meal intake. In addition, the insulin concentrations in the EM trials were significantly lower than those in the EP trials at 180 min (*P* = 0.034) after meal intake. A three-factor ANOVA revealed a significant trial-time interaction (*P* = 0.001). Post-hoc tests revealed that the GIP concentrations in the MM trials were significantly lower than those in the MP trials at 120 min (*P* = 0.001) and 180 min (*P* = 0.010) after meal intake. In addition, the GIP concentrations in the EM trials were significantly lower than those in the EP trials at 120 min (*P* = 0.024) after meal intake. For serum TAG and NEFA concentrations, a three-factor ANOVA revealed a significant timing × time interaction (*P* = 0.001). However, there were no significant differences in serum TAG and NEFA concentrations between MLE and placebo in either the morning or evening trials.

## Discussion

To our knowledge, this study is the first report on effects of the time of the day of MLE intake on postprandial glucose metabolism in young adults. The main finding was that acute intake of MLE in the evening, but not in the morning, significantly suppressed the iAUC and postprandial glucose concentrations. Our results reveal that MLE intake in the evening was effective in improving glucose tolerance, which is associated with diabetes development. They also provide novel evidence regarding the timing of MLE intake to elevate its antidiabetic effect from a chrononutrition perspective.

Increasing evidence suggests that the postprandial glucose response to dinner is higher than that to breakfast [8, 9, 11]. A recent meta-analysis also revealed that glucose tolerance was poorer at night than during the day [24]. Our results are consistent with these results. These differences may be attributed to a reduction in insulin sensitivity in the evening and decreased insulin secretion [11, 25]. Additionally, insulin concentrations in the morning trials, 30 min after meal intake, were significantly higher than those in the evening trials. Moreover, NEFA concentrations were significantly higher in the evening trials than in the morning trials. By inducing insulin resistance, elevated NEFA levels play a pivotal role in the development of type 2 diabetes [26, 27]. Our results show that glucose tolerance is worse at night, which is caused by insulin sensitivity and functions regulated by circadian rhythms.

DNJ, derived from MLE, has been used in several countries to treat diabetes because of its effects on lowering blood glucose concentrations. Several studies have reported that MLE that contains DNJ possesses glucose-lowering and antidiabetic properties [13–15]. However, no information is currently available regarding the timing effects of MLE intake on postprandial glucose metabolism and insulin response in humans. In this study, we showed that MLE intake, especially in the evening, decreased the iAUC and reduced the elevation of postprandial glucose concentrations. One reason for these decreases only in the evening trials may be attributed to diurnal variation in glucose tolerance. As postprandial glucose concentrations in the evening trials were higher than those in the morning trials, consuming MLE in the evening, when glucose tolerance worsens, may enhance its glucose-lowering effects. Carbohydrate-digesting enzymes (α-amylases and α-glucosidases) are also thought to be regulated by the circadian clock. DNJ intake inhibits α-glucosidases in the small intestine and blocks glucose absorption in the small intestine [28, 29]. DNJ is not only a potent inhibitor of intestinal α-glucosidases but also modulates hepatic glucose metabolism and gluconeogenesis by upregulating or downregulating the mRNA expression of enzymes in diabetic mice [29]. Altering the timing of MLE intake may change these enzymes, and modify its glucose-lowering effect.

There are some clinical implications from our results. First, regarding the dose of MLE, we decided to administer 6.0 mg of DNJ with a test meal in this study based on previous studies [17, 30]. Thus, the dose of MLE in this study is similar to that naturally present in food and beverages (i.e., green tea containing MLE or DNJ) and that consumed in daily life. Second, many epidemiological studies have reported an association between meal timing and health and clinical outcomes (i.e., BMI, fasting and postprandial glucose metabolism, and blood pressure) [31, 32]. Increasing evidence has shown an association between an evening chronotype (i.e., young and middle-aged population) and an increased risk of obesity, CVD, and diabetes [33]. In general, the evening chronotype is associated with larger meals later in the day and delayed food intake due to a later awaking time. Although dinner time varied between from 17:00 to 24:00 in previous studies, dinner caused a higher postprandial glucose response than that caused by breakfast [8, 11, 22]. Thus, the effectiveness of MLE in lowering glucose levels may be optimal if taken at dinner time. Lastly, our findings are useful for designing chrononutrition studies focusing on the effects of timing of the intake of antidiabetic foods on postprandial glucose levels.

## Conclusion

Our findings demonstrated that acute MLE intake in the evening, but not in the morning, exhibited glucose-lowering effects, which may be a useful tool for the prevention of diabetes and obesity when accumulating these effects.

## Supplementary information

Fig. S1

CONSORT 2010 Checklist

## Data Availability

The data that support the findings of this study are available from the corresponding author upon reasonable request.
