# Supplementary material for: Effects of the timing of acute mulberry leaf extract intake on postprandial glucose metabolism in healthy adults: a randomised, placebo-controlled, double-blind study
Source: Eur J Clin Nutr. 2023 Jan 17;77(4):468–73. doi: 10.1038/s41430-023-01259-x (PMC10115625; doi:10.1038/s41430-023-01259-x)
Supplement: Supplementary file 1 — Fig. S1 [file 41430_2023_1259_MOESM1_ESM.doc]

**
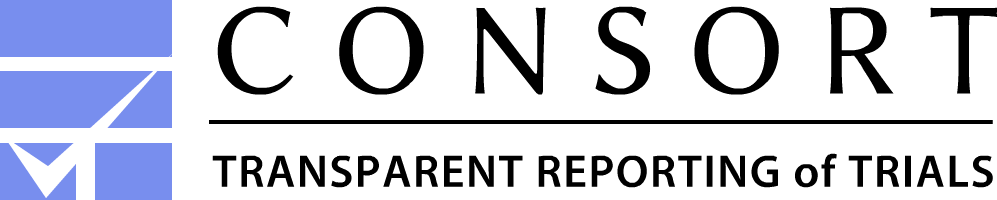
**

**CONSORT 2010 Flow Diagram**

**Analysis**

**Enrollment**

**Allocation**

Assessed for eligibility (n=13)

Excluded (n=1)

♦  Not meeting inclusion criteria (n=0)

♦  Declined to participate (n=1)

♦  Other reasons (n=0)

MP (Morning Placebo)

(n=12)

Randomized, placebo-controlled, double-blind, counterbalanced crossover trials (n=12)

MM (Morning MLE)

(n=12)

EP (Evening Placebo)

(n=12)

EM (Evening MLE)

(n=12)

Analysed

(n=12)

Analysed

(n=12)

Analysed

(n=12)

Analysed

(n=12)
